# Turbulence in surgical suction heads as detected by MRI

**DOI:** 10.1051/ject/2023015

**Published:** 2023-06-28

**Authors:** Gunnar Hanekop, Jost M. Kollmeier, Jens Frahm, Ireneusz Iwanowski, Sepideh Khabbazzadeh, Ingo Kutschka, Theodor Tirilomis, Christian Ulrich, Martin G. Friedrich

**Affiliations:** 1 Department of Anesthesiology, Intensive Care, Emergency Medicine, Pain Therapy, University Medicine, Georg-August-University Robert-Koch-Strasse 40 37075 Goettingen Germany; 2 Max-Planck-Institute for Multidisciplinary Sciences Am Faßberg 11 37077 Goettingen Germany; 3 Department of Heart-Thoracic- and Vascular-Surgery, University Medicine, Georg-August-University Robert-Koch-Strasse 40 37075 Goettingen Germany

**Keywords:** Suction, Blood damage, Hemolysis, Turbulence, Magnetic Resonance Imaging

## Abstract

*Background*: Blood loss is common during surgical procedures, especially in open cardiac surgery. Allogenic blood transfusion is associated with increased morbidity and mortality. Blood conservation programs in cardiac surgery recommend re-transfusion of shed blood directly or after processing, as this decreases transfusion rates of allogenic blood. But aspiration of blood from the wound area is often associated with increased hemolysis, due to flow induced forces, mainly through development of turbulence. *Methods*: We evaluated magnetic resonance imaging (MRI) as a qualitative tool for detection of turbulence. MRI is sensitive to flow; this study uses velocity-compensated T1-weighted 3D MRI for turbulence detection in four geometrically different cardiotomy suction heads under comparable flow conditions (0–1250 mL/min). *Results*: Our standard control suction head Model A showed pronounced signs of turbulence at all flow rates measured, while turbulence was only detectable in our modified Models 1–3 at higher flow rates (Models 1 and 3) or not at all (Model 2). *Conclusions*: The comparison of flow performance of surgical suction heads with different geometries via acceleration-sensitized 3D MRI revealed significant differences in turbulence development between our standard control Model A and the modified alternatives (Models 1–3). As flow conditions during measurement have been comparable, the specific geometry of the respective suction heads must have been the main factor responsible. The underlying mechanisms and causative factors can only be speculated about, but as other investigations have shown, hemolytic activity is positively associated with degree of turbulence. The turbulence data measured in this study correlate with data from other investigations about hemolysis induced by surgical suction heads. The experimental MRI technique used showed added value for further elucidating the underlying physical phenomena causing blood damage due to non-physiological flow.

## Introduction

Maintaining adequate oxygen levels, determined by hemoglobin concentration (Hb) and/or hematocrit (HCT), is critical during surgery. Reduced values are significantly associated with increased perioperative mortality [1, 2], although the lower limits are not clearly defined [3–9]. On the other hand, allogenic transfusions may contribute to an increased perioperative mortality up to 70% [10, 11]. Cardiac surgery is often associated with intraoperative losses of more than 9% of the circulating blood volume [9, 12], resulting in transfusions in more than 50% of procedures [13], accounting for 15–20% of all perioperative allogenic transfusions [1, 14, 15].

Patient blood management (PBM) [12] recommends maintaining the blood volume by rescuing the patient`s own red blood cells (RBCs) from the surgical field [16]. In cardiac surgery this is done by direct suctioning of shed blood into the venous reservoir of the extracorporeal circulation (ECC) via filter systems, or by collecting and processing of wound blood with cell salvage devices [17–23]. Both procedures are safe, cost-effective [14, 24], useful [25–31], and have become routine in most cardiac centers [32–35]. This can result in a reduction of all allogenic transfusions by nearly 40% [36–38], provided that the integrity of the corpuscular cells can be preserved using these techniques [39].

Traumata to blood components during ECC, mechanical circulatory support (MCS), and suction devices [40–45], may among others be triggered by supra-physiological shear stress induced by turbulence and/or bubble formation [46–49]. This flow is described as random, chaotic, irregular and multi-scale, with high vorticity and pressure and velocity fluctuations on large time and length scales (~10^12^) [28]. The direction and velocity field of turbulence can only be described mathematically by statistical parameters. High friction losses occur due to momentum transport diagonally to the main flow. Many experimental and numerical studies have shown a general increase in hemolytic activity in turbulent compared to laminar conditions [50], although the underlying mechanisms are not fully understood [51, 52].

The negative pressure required to aspirate from the surgical field can be generated by roller pumps or central vacuum. Which of these sources is more suitable and causes the least amount of trauma to the corpuscular cells and/or plasma proteins is still an unsolved problem [53–57].

In previous experiments, we have shown that different geometries of suction heads result in different flow characteristics and other physical properties with different degrees of blood damage [58]. This has also been demonstrated by different noise distributions in the frequency domain [59]. Noise is significantly induced by and correlated with turbulence [60–62]. Turbulent blood flow occurs in cardiovascular devices and negatively affects the integrity of the blood and its components due to microscale flow fields [51, 63]. This assumption is supported by the findings of Kameneva et al. [64], who confirmed that the mechanisms of flow-induced hemolysis show fundamental qualitative and quantitative differences in laminar and turbulent flow. In this study, we hypothesized that different suction head geometries would cause different degrees of turbulence. Using magnetic resonance imaging (MRI), we have tried to capture these changes. This approach has not been the subject of previous research.

## Materials and methods

In this study, MRI is evaluated as a qualitative tool for the spatial resolution of areas of turbulent flow in a variety of suction head geometries. MRI provides a wide range of image contrasts and can be sensitive to flow. While there exists a method for quantitatively assessing flow velocities called phase contrast (PC) MRI [65], it only works reliably in laminar flow conditions [66]. However, in the presence of turbulence, the PC MRI signal can be degraded to the point of complete extinction [67], making the application of standard PC MRI to surgical aspiration impractical, as we can expect this to be associated with turbulent flows in most circumstances. For this reason, we relied on velocity-compensated T1-weighted 3D MRI. This modality is sensitive to large fluctuations in flow acceleration and is therefore a potential marker for turbulence.

**Figure 1:**
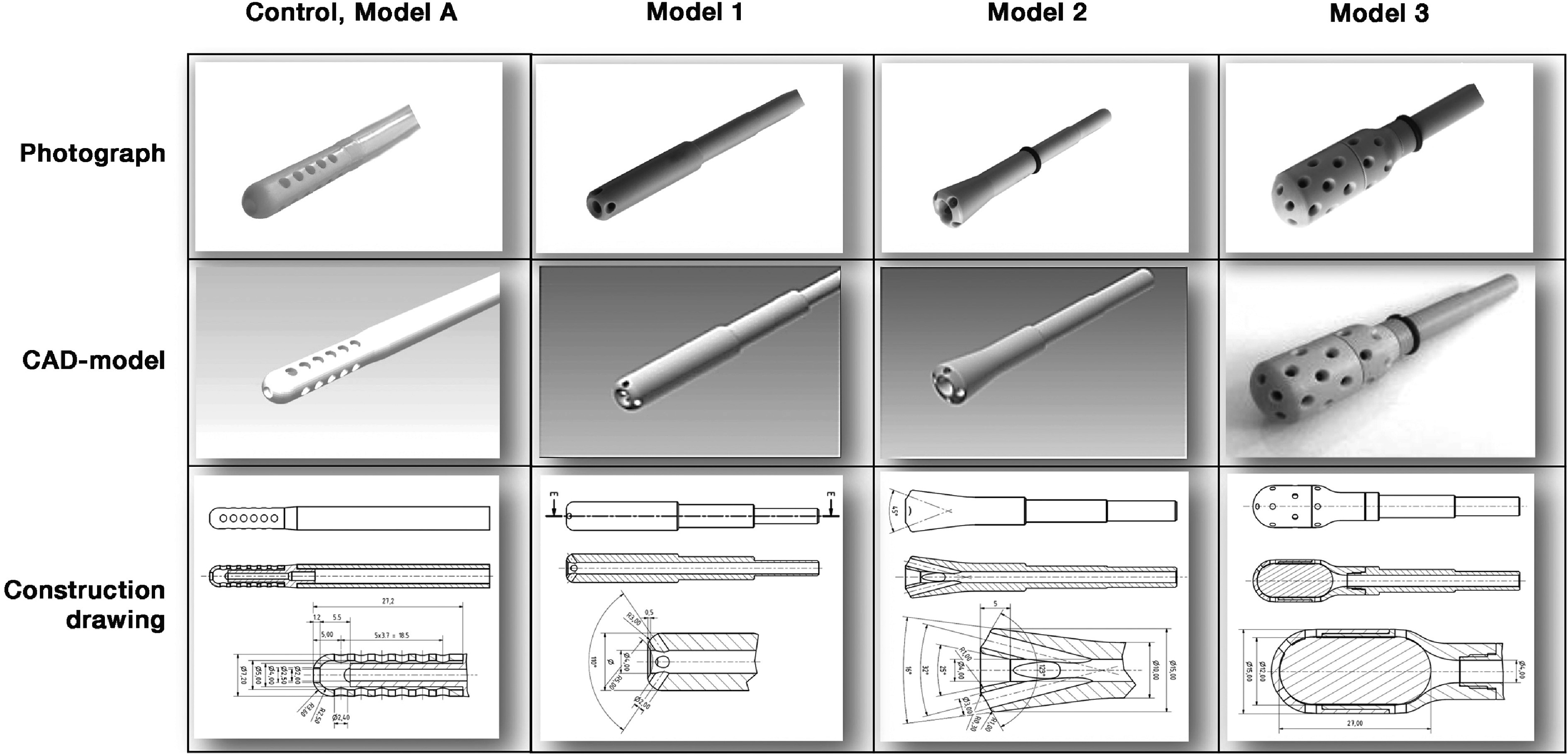
Photographs and computer assisted design (CAD) models and drawings of suction heads under investigation (adapted from [59]).

To determine the flow characteristics of different suction head designs, we performed volumetric MRI scans on the four different models (three of which self-developed; cf. Figure 1) at varying flow rates (0, 250, 500, 750, 1000, 1250, 0 mL/min). The control suction head Model A (Hex Handle Adult Sump Sucker, NovoSci, Eder&Eder, Vienna, Austria) is the standard commercial model used in our hospital, the modified in-house designs (Models 1–3; 3D-printed with Surgical Guide Resin RS-F2-SGAM-01, Formlabs, Boston, USA) are based on preliminary experimental tests in an in vitro model with hemolysis (data not shown here) as well as mechano-acoustic measurements [59].

The MRI technique used in this study is based on the theory of PC MRI and exploits the effect of turbulence-induced signal reduction (“dephasing”) to detect the presence and existence of turbulence.

The complex MRI signal phase *φ* is controlled by applying user-controllable magnetic field gradients *G*(*t*). The phase of the MRI signal is then given by

(1) $$\varphi =\gamma \;\int_{0}^{\mathrm{TE}}G\left(t\right)\;x\left(t\right)\;dt,$$

where *γ* denotes the gyromagnetic ratio for hydrogen nuclei, TE the echo time, and *x*(*t*) the trajectory of an excited ensemble of spins.

Using a Taylor expansion $x\left(t\right)\approx x_{0}+\upsilon t+\frac{1}{2}{\mathrm{at}}^{2}\ldots \;$ and neglecting the higher terms, it is possible to derive the phase evolution as a function of the position *x*_0_, the velocity υ and the acceleration *a* of the moving spins:

(2) $$\varphi \approx \gamma \;\int_{0}^{\mathrm{TE}}G\left(t\right)\;\left(x_{0}\;+\;\upsilon t\;+\;\frac{1}{2}\;at^{2}\right)\;dt,$$

The zero, first and second magnetic gradient moments *M*_0_ *, M*_1_ *,* and *M*_2_ are adjustable by gradient design and follow:

(3) $$M_{n}=\gamma \;\int_{0}^{\mathrm{TE}}G\left(t\right)\;t^{n}dt.$$

The phase contribution of the moving spins can be approximated as follows:

(4) $$\varphi =M_{0}x_{0}+M_{1}\upsilon +M_{2}\frac{a}{2},$$

*M*_0_ is used for spatial encoding and echo formation, while *M*_1_ and *M*_2_ are not explicitly defined in conventional MRI of static tissue. *M*_0_ is set to zero and *M*_1_ is adjusted for velocity quantification, while any acceleration *a* and thus *M*_2_ are neglected, in PC MRI applicable to laminar flow. While this approach yields a phase proportional to the velocity *φ* ∝ *υ*, in the presence of turbulent flow, the size of the complex MRI signal decreases when different velocity values are present within the same image voxel (i.e., a three-dimensional pixel; [68]). This is due to the overlapping of different velocity components with correspondingly different phase shifts. This leads to mutual signal cancellation, also known as *dephasing*. This effect is exploited in Dyverfeldt et al. [69] to image turbulence and quantify the intravoxel velocity standard deviation, assuming Gaussian velocity distributions.

Unfortunately, two factors limit the application of this method to the surgical suction heads we were interested in: (i) computational fluid dynamics (CFD) simulations (data not shown) predict high velocity values above 4 m/s, promising high fluctuations and thus pronounced signal loss due to phase cancellation, and (ii) since suction head size requires small voxel sizes, the MRI signal per voxel becomes intrinsically low. For these reasons, this work does not image turbulence based on variations in velocity υ, but instead exploits variations in acceleration a. By using flow compensating gradients that yield *M*_0_ = *M*_1_ = 0, thus avoiding phase cancellation due to variable velocities υ, the method used here aims to potentiate higher MRI signal intensities. However, any temporal or spatial variation in acceleration *a* will result in MRI signal reductions indicative of turbulence if the sensitivity is *M*_2_ ≠ 0. Therefore this study is based on acceleration sensitive MRI.

Volumetric MRI images were acquired for all suction heads whilst operating in water (test liquid) at flow rates up to 1250 mL/min. In previous experiments, water was replaced by a glycerol (C_3_H_8_O_3_)/water mixture with a viscosity close to that of blood (4.5 mPa s), where the MRI data showed no significant change in flow behavior compared to purified water. For practical reasons, we therefore decided to continue with the use of water as a test medium. T1-weighted images of each suction head and its water-filled environment were taken using a spoiled gradient echo sequence.

Flow compensation was enabled in all three directions to minimize signal loss due to velocity induced phase dispersion (i.e. resulting in *M*_0_ = *M*_1_ *= 0*, *M*_2_ ≠ 0). Each measurement covered a 3D volume of 30 × 40 × 23 mm^3^ with an isotropic spatial resolution of 0.25 mm. Other MRI parameters included a flip angle of 6°, an echo time TE of 8.62 ms, a repetition time of 16 ms, and three averages. This resulted in a total acquisition time of 516 s for each MRI scan.

**Figure 2:**
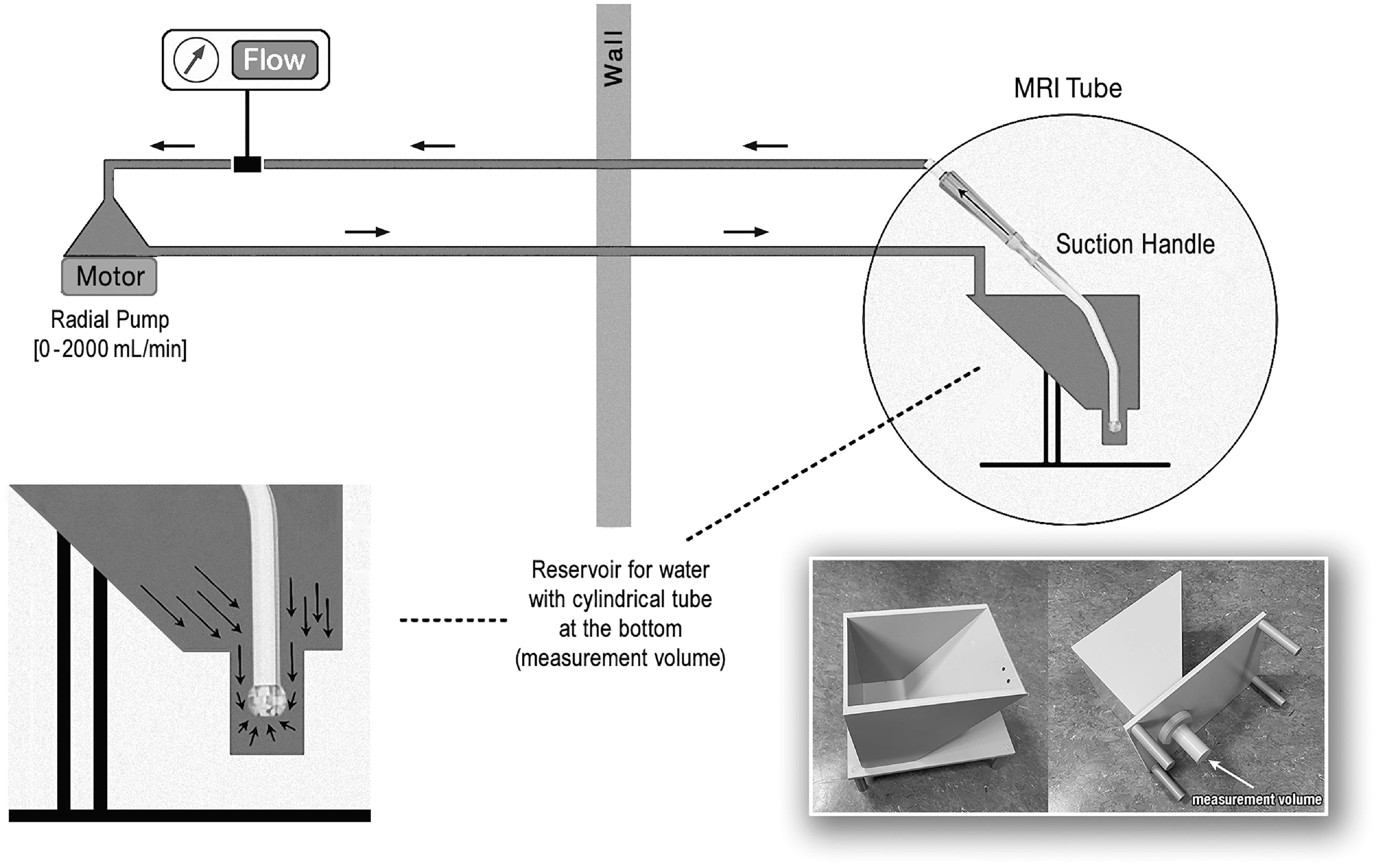
Experimental setup including custom-made water reservoir for MRI measurements.

The experiments were performed on a 3T MRI system (Magnetom-Prismafit, Siemens Healthineers, Erlangen, Germany) using a 4 cm loop coil for signal acquisition. Each suction head was placed in a customized MR-compatible water reservoir (see Figure 2, bottom right), allowing close positioning of the receiver coil. To prevent pulsatile flow, each suction head was connected to a standard radial pump (BioMedicus 550 radial pump, Medtronic, Minneapolis, USA; Sarns-Terumo radial pump head with Sarns radial pump adapter for BioMedicus 540/550, Terumo, Shibuya, Japan) located outside the MRI room. A transit time ultrasonic flowmeter (HT110 and flow-probe H7XL, Transonic Systems Inc., Ithaca, NY USA) was used to control flow constancy during experiments. Starting from zero flow, the pump rate was increased in increments of 250 mL/min to a maximum of 1250 mL/min. In order to minimize potential errors due to temperature changes, the initial zero measurement was repeated at the end of each series resulting in a total of seven MRI scans for each suction head.

All MR images were denoised using a non-local mean-based image filter [70] in a post-processing step. Images with flow-induced MRI signal reduction were then calculated by subtracting the magnitude images acquired with and without flow. A linear combination of the two zero-flow measurements from each suction head was established as the reference. This average was then used to normalize the resulting difference maps to obtain relative signal differences. These relative difference 3D data sets show areas of flow-induced signal loss. These areas are representative of turbulent flow.

## Results

**Figure 3:**
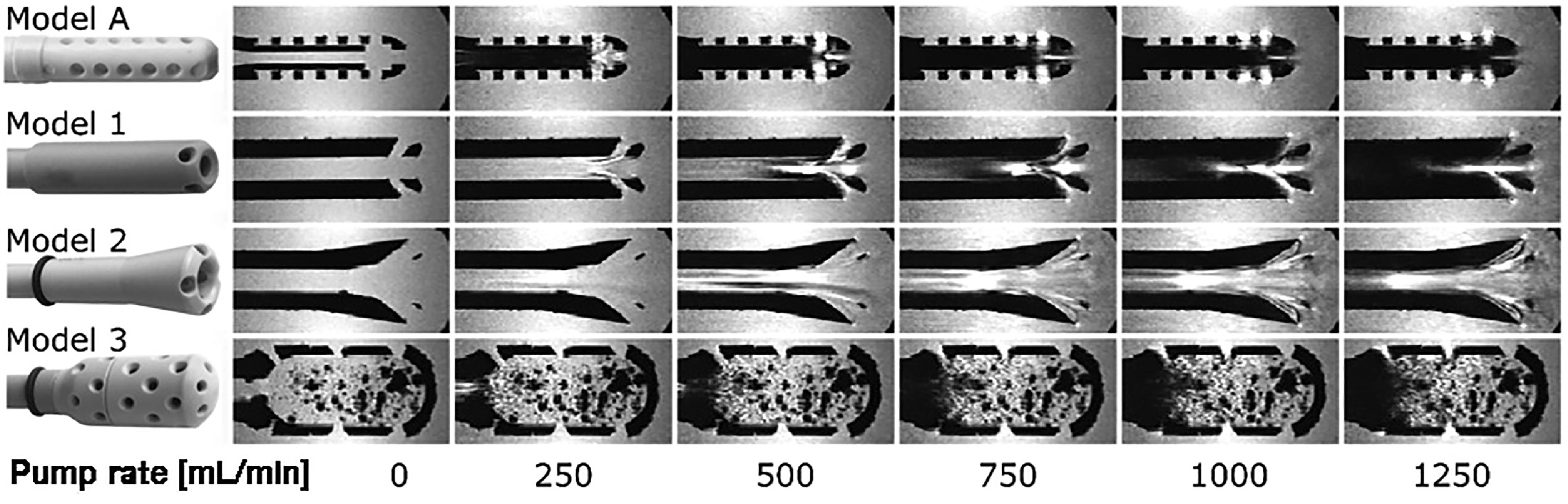
T1-weighted images of surgical suction heads in water. Increasing flow rates introduce signal void indicating areas of turbulent flow.

Measurements were performed under stable flow conditions, obtaining three-directional acceleration sensitive data sets for all four models. Figure 3 shows selected images of the T1-weighted MRI data for all suction heads and pump rates. These are shown as cross-sections of each 3D data set.

In a post-processing step, the relative signal reduction or even complete loss due to turbulence was determined.

**Figure 4:**
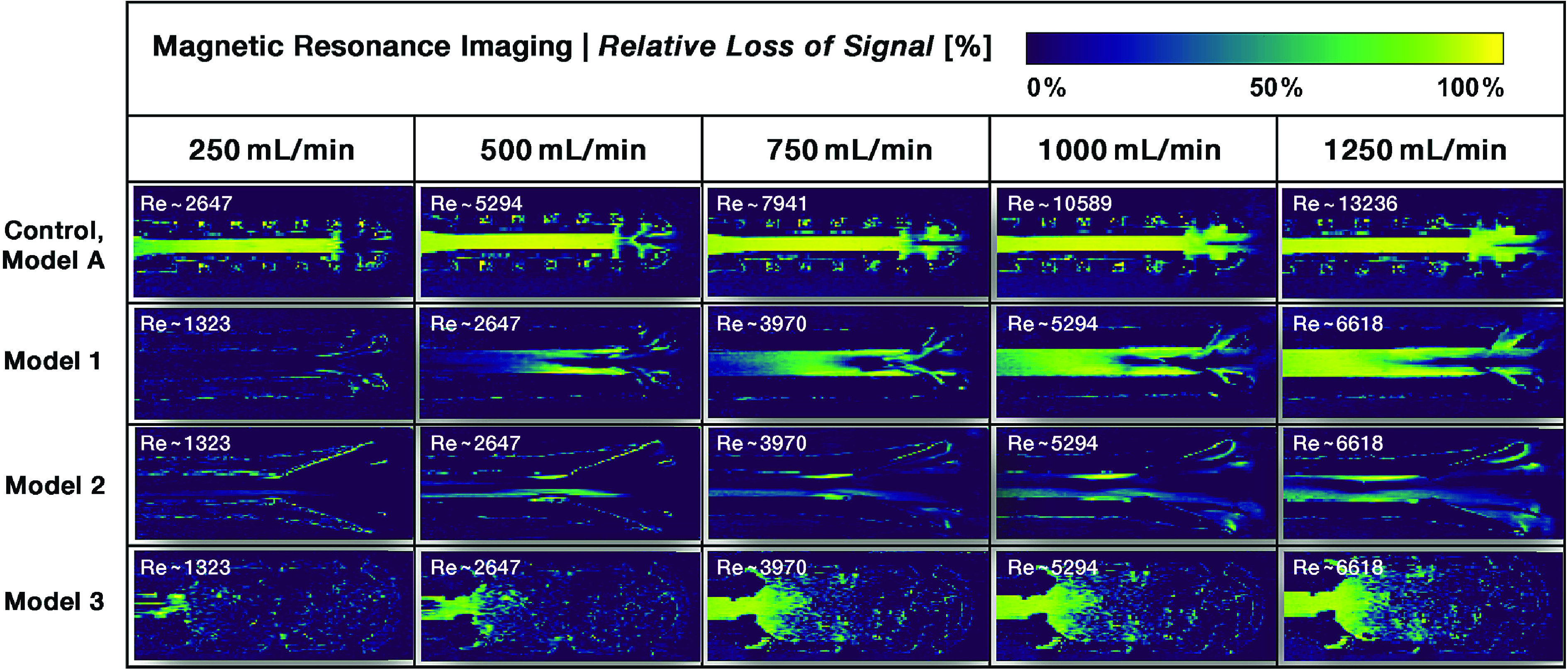
MRI signal loss in surgical suction heads calculated from images in Figure 3 with corresponding Reynolds number (Re).

Figure 4 depicts the post-processed color-coded pixel values representing the relative signal attenuation due to turbulent flow – scaled by color change from blue (no attenuation) to green to light yellow (complete loss) – for the different suction heads at each flow rate and measurement.

The relative difference maps highlight areas of turbulent flow and allow a comparison of the different models.

In control Model A, laminar flow can be seen at the entrance to the suction head, both in the central channel as well as in the first row of side holes, at a flow rate of 250 mL/min (Reynolds number ~2600), corresponding to a velocity of approximately 133 cm/s. At the point of intersection of these two flows, turbulence is present. This results in flow separation from the wall layers due to additional friction and increased resistance caused by the inflow through the side holes (similar to a manifold or bend). Transverse secondary flows are superimposed on the laminar primary flow due to centrifugal forces. This complicated mixed flow significantly influences the flow profile of the subsequent outlet section. As the flow rate increases, more and more turbulent phenomena is detected in the inflow region. However, even at the highest flow rate of 1250 mL/min (Reynolds number ~13,250), there is still laminar flow in the center of the inflow region.

In the three modified models only Model 3 shows traces of turbulence at the entrance of the central channel at the lowest flow rate (Reynolds number ~1320, velocity about 33 cm/s) while the other two models consistently show laminar flow in this area. With increasing flow rate (Reynolds number >2600) a loss of signal is observed in Model 1 after the entrance of the suction head side holes. However, the movement of the fluid in the central suction channel remains laminar and seems to stabilize the flow in the following segments. As the flow rate increases, this effect becomes more pronounced. It appears that the laminar flow in the central part of the suction channel is no longer able to suppress turbulence from the side holes at 1000 mL/min, corresponding to a velocity of ~133 cm/s. Thus, the laminar flow starts to become turbulent. Similar to Model 1, Model 2 shows only discrete evidence of turbulence at low Reynolds numbers. At a flow rate of 500 mL/min, slight signs of detachment can be seen at the confluence of the side holes with the main suction channel. However, these signs of turbulence disappear very quickly. As the flow rate increases, these detachments become larger in size, while the central flow region appears to remain laminar up to the highest flow rate measured.

Due to the complex geometry of Model 3, only the entry of the flow into the central suction channel is considered. Even at the lowest Reynolds number of ~1320 with a corresponding flow rate of 250 mL/min, small areas of turbulence can be seen. Above 750 mL/min, complete MRI signal cancellation is observed in the central suction channel and its upstream funnel-shaped area, indicating a strong presence of turbulence.

**Figure 5:**
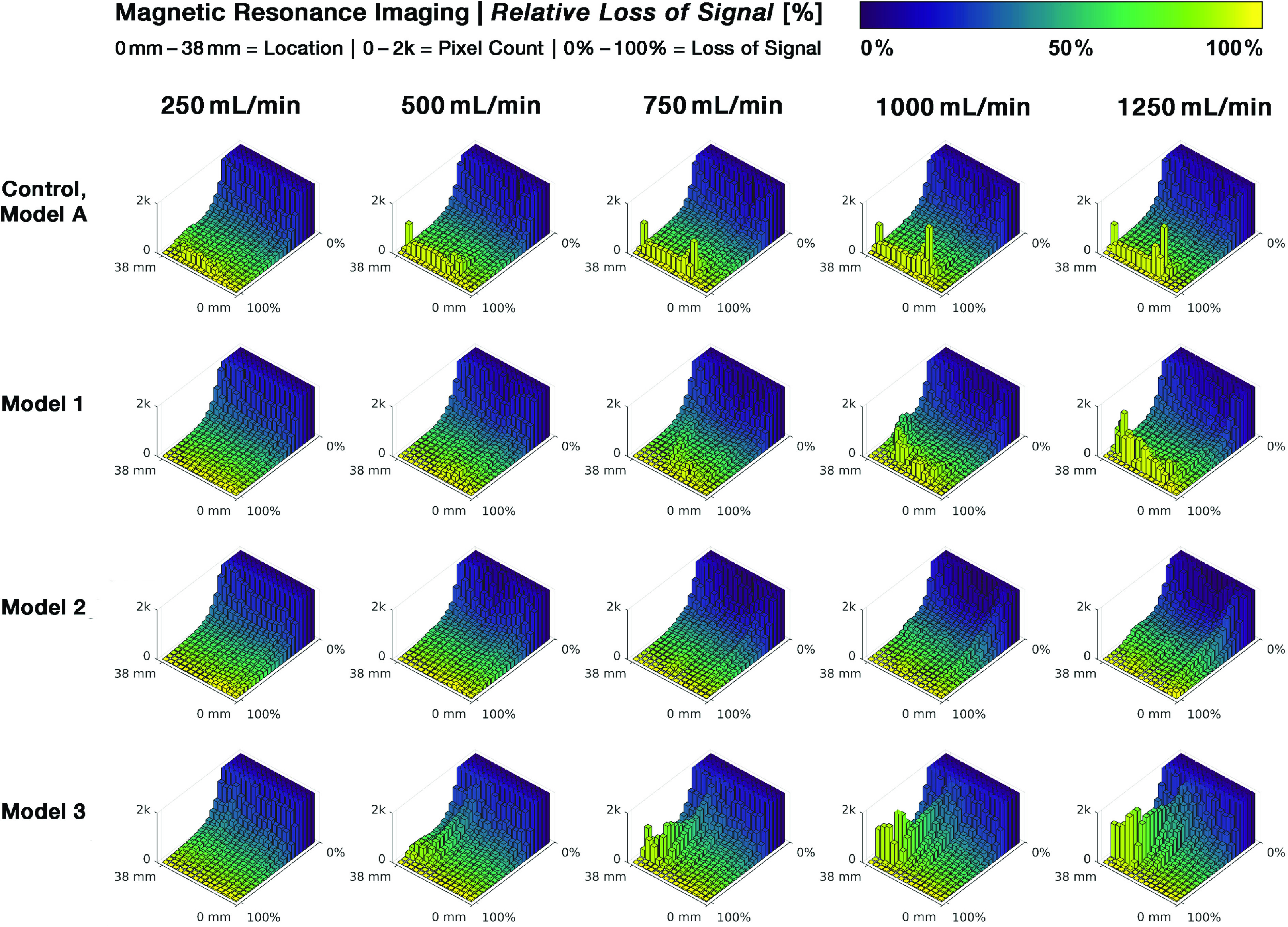
Histograms of relative signal loss images from Figure 4. Distinct yellow bars relate to the occurrence of signal loss.

As an alternative to the graphical representation of signal loss in Figure 4, 3D histograms of the images in Figure 5 further summarize differences in relative signal cancellation along the suction head tips. Areas of turbulent flow are indicated by yellow peaks representing high signal loss in the histograms.

Model A shows an increasing bimodal signal cancellation curve for all measured velocities. In Model 2, no increase in signal attenuation is detectable at higher flow rates, indicating more optimized flow conditions. In Models 1 and 3, it is only the closest parts of the suction duct that show an increase in turbulence as the flow rate increases.

## Discussion

This study compares the flow performance of geometrically different surgical suction heads using in-vitro acceleration-sensitized 3D MRI. The specific MRI contrast used allows a qualitative visualization of turbulent areas in the inner channels of the different models as a function of flow rate.

**Figure 6:**
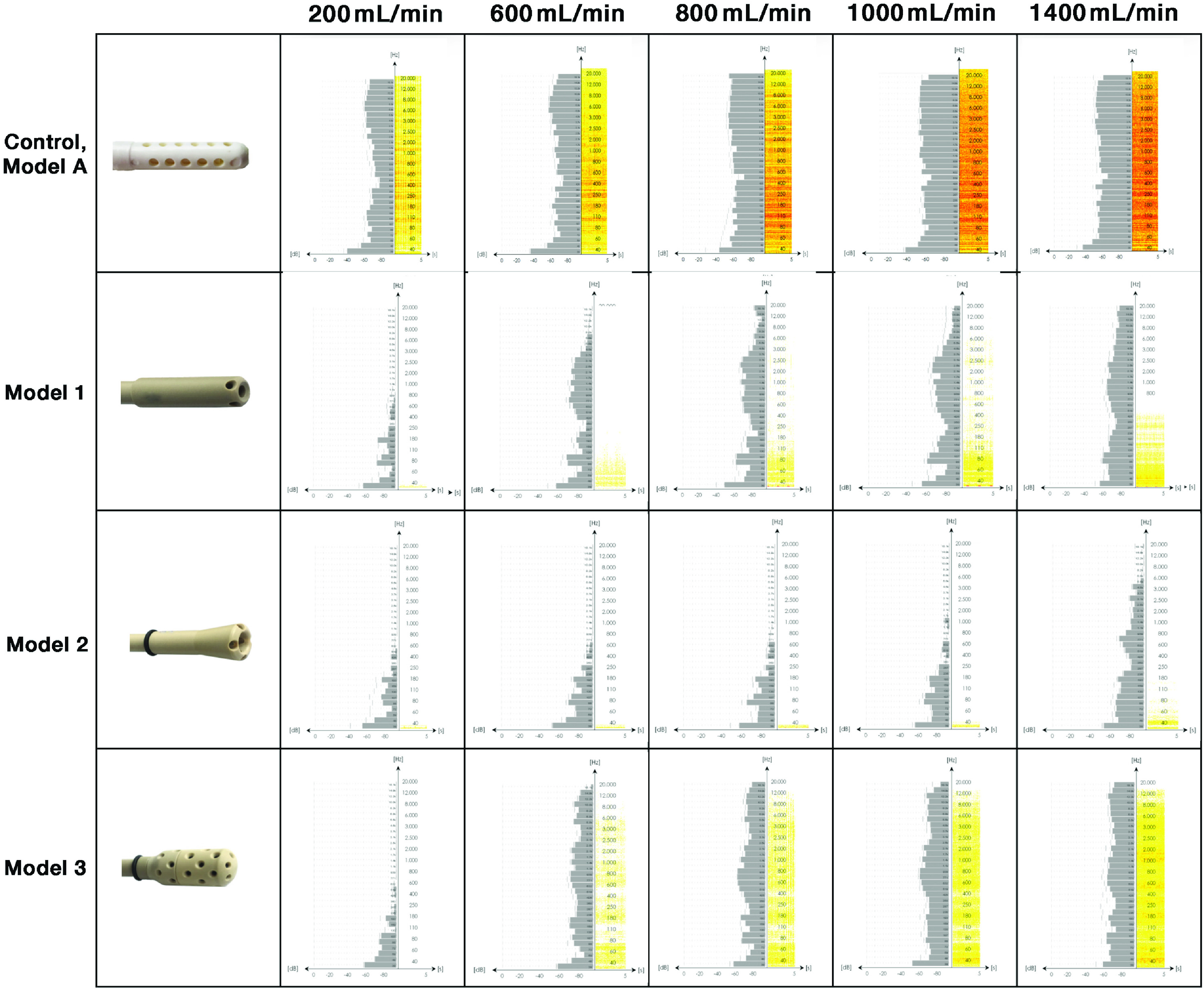
Noise-maps of suction heads studied (adapted from [59]).

The qualitative results agree well with preliminary quantitative CFD modelling data for standard Model A and Models 1 and 2 (unpublished data). However, due to possible *inlet length effects*, it is not evident that the flow profile within the suction head is complete [71, 72]. Our experimental MRI study realized higher flow rates than a corresponding CFD simulation (data not shown), that was limited by computational requirements. Both aspects highlight the added value of experimental MRI for further elucidating the underlying physical laws of nature for blood damage due to non-physiological flow. But we cannot infer resulting shear or other damaging forces and potential RBC compromise. However, the MRI results correspond with noise maps [59] obtained from the same geometrically different suction heads at similar flow rates (see Figure 6).

We can only speculate about the mechanisms responsible for the development of turbulence in the suction heads and its potential to cause blood damage. However, general fluid dynamics findings allow some for conclusions. For example, although this view has not gone unchallenged [73–76], Blackshear et al. [77] have pointed out that in addition to turbulence there are several factors in pump circuits that can damage blood components: e.g. pressure drops, shear stress and other forces. These forces are thought to exert a disruptive, borderline damaging potential on corpuscular and plasmatic blood components, the latter, particularly von Willebrand factor (vWF), being cleaved by ADAMTS-13 under turbulent flow [78, 79].

The magnitude and duration of these serious stresses have been associated with hemolysis and inflammatory response [48, 80–85], although it has also been shown that for the same scalar flow stress, the level of erythrocyte membrane tension is highly variable [51]. There is evidence that air aspiration and turbulent flow development appear to be additional stresses on the blood components [76, 86], unfortunately we were unable to investigate this because of the fundamental limitations of the MRI technique in multi-phase flow (see chapter Limitations). Wright and Sanderson [44] reported that the percentage of free Hb (seen as a surrogate for blood damage) in cardiotomy suction was significantly higher in the cardiotomy reservoir than in the circulating blood in the oxygenator, a finding that was subsequently confirmed [86–88]. Wright [89] attributed this to the combined aspiration of blood and air, referring to Rygg [90] who concluded, that for the same physical force (suction), air flows at a much higher velocity than blood, resulting in significant turbulence-induced shear forces, a finding supported by other authors [91].

Hemolytic damage to blood components by mechanical stress and exposure time is often described by an empirically based exponential correlation, the *power law model* [87, 88, 92, 93]. Although widely used, this model is not always accepted due to the underlying assumptions and constants involved [94].

It has been shown that different corpuscular and plasmatic components of the blood tolerate mechanical stress very differently, but that the more pronounced the negative pressure and the longer the exposure time, the greater the damage to be expected [95–100]. This finding is not limited to cardiotomy suction. It also applies to the use of “cell-saving” and “venting” drainage systems. The results of the model calculation by Wright [89] were confirmed by the findings of Paul et al. [95], who concluded that under routine clinical conditions it may be almost impossible to keep the shear forces during cardiotomy and cell-saving suction below the known tolerable shear limits for red cells of about 50 N/m^2^. This implies that some degree of traumatic damage to red blood cells appears to be unavoidable, and that for this reason any factors leading to additional forces on the blood during aspiration must be avoided.

From the MRI data reported here, it is reasonable to surmise that – assuming no air admixture and Newtonian flow behavior – impulse and bending effects inside the suction head ducts play a significant role in the damage potential of the suction process during surgery. This is due to the introduction of secondary forces into the downstream flow, such as centrifugal forces and increased shear stress in bends, with pressure losses and distorted velocity profiles [101, 102]. These effects can be detected at the entrance of the side holes located at the tip of the suction heads. This is also confirmed by the histograms of MRI data for Models A and 1 (see Figure 5), where higher degrees of measured signal loss are concentrated at the tips of the suction heads. Flow perturbations due to pipe bends have been shown for distances up to 50D from the central plane of the bend [103]. This is longer than our measurement range. Bending effects are quite likely to occur at the entrance of side holes, but the importance of laminar flow in the center of the pipe for the flow behavior in the following parts of the suction head can be deduced from the MRI data of Models 1 and 2, where turbulence, probably induced by bending due to momentum effects, was terminated by the laminar central flow. This may be caused by work induced by acceleration, which may force the detached streamlines to be reattached to the channel wall, thus allowing laminar flow to resume. This assumption is supported by the findings of Schneider et al. [104] regarding the “edge of chaos”, which separates perturbations in the flow that decay back to a laminar profile from those that prevail and induce turbulence. Only perturbations of sufficient strength trigger the transition of the flow field to turbulence. Many experimental studies have shown a surprising spatially discontinuous behavior of the transitional flow in pipes, such as the existence of turbulent patches separated by regions of laminar flow for a range of flow rates beyond those that would be expected to be necessary for the generation of turbulence [105, 106], only at higher speeds the entire flow may become turbulent, as can be seen in Model 1 with increasing flow rates (>1000 mL/min). Further studies must show whether these changes can also be observed in non-Newtonian fluids and with air admixture or if they are even aggravated under these conditions.

In total, this MRI study confirms data we have previously obtained by measurement of the noise distribution in these same suction heads (see Figure 6; [59]). From both studies it can be concluded that the damage potential of the control suction head Model A is expected to be much higher than that of the modified models. This is particularly true under clinical conditions when air is aspirated with blood. This creates a “slug flow” with a significantly increased potential for damage [107].

The avoidance of multiple side holes with perpendicular confluences due to these additional openings and any narrowing of the inner channel are suggested reasons for the more favorable behavior of the modified suction heads.

Kameneva et al. [64] confirmed that the mechanisms of flow-induced hemolysis are fundamentally different in laminar and turbulent flow. Turbulent tube flow at a Reynolds number of 5100 results in a six times higher hemoglobin release compared to laminar flow yielding the same mean wall shear stress. These findings are consistent with the results of the present study.

Historically, proposals to reduce hemolysis during cardiotomy suctioning have fallen into three broad groups:

- Efforts to avoid cardiotomy suction entirely, either by using closed, miniaturized CPB systems, by eliminating re-transfusion of shed blood [108–114], or by replacing cardiotomy suction with cell saving [31, 100, 115–120].
- Attempts to modify the suction heads or whole systems in order to minimize the resulting abnormal flow changes, shear forces, and air admixture in cardiotomy suction [87, 91, 121–125].
- “Sucker discipline”, a behavioral solution mentioned by Riley [126], who proposed that surgeons should suck from pools of blood rather than from surfaces, because surface “skimming” increases hemolysis [39]. Although washing processes in cell salvage can almost entirely eliminate all free hemoglobin, destroyed erythrocytes cannot be re-transfused because they are completely discarded.

Our results show a strong correlation between flow velocity and signal reduction, making the applied MRI technique and the resulting image contrast suitable for qualitative imaging of turbulence and thus blood impairment. The different damage potential is also supported by theoretical considerations and is in good agreement with data obtained for different shapes of suction tips by introducing the dimensionless Q-factor in a paper by Iwanowski et al. [58]. A higher Q-factor means that the tip will perform worse in terms of hemolysis. The finding of the same Q-factor [0.94] for both Model 1 and Model 2 is due to the fact that in addition to the turbulence behavior, which is quite different between these modified suction heads, other factors are included in the calculation of Q, so that relevant harmful or protective parameters may lead to compensatory effects.

Taken together, the present MRI data show a clear relationship between suction head geometry and amount of turbulence generated. Since turbulence appears to be responsible for the development of hemolysis in MCS devices and surgical suction, our results suggest that geometric modification of these systems is a useful way of amelioration. It could help to reduce blood trauma and hemolysis and may thus reduce the need for allogenic blood by saving more erythrocytes for re-transfusion.

In line with previous findings regarding acoustic benefits and the theoretical Q-factor [58, 59], the new modified models appear to be favorable compared to the commercial suction device used as a control. This provides strong evidence that optimizing the suction head geometry may be a step towards improving the quality of blood collected and salvaged during surgical procedures, but the different test conditions (newtonian fluid, no air admixture) must be taken into account when interpreting the results. However, in-vitro testing of these different suction heads for hemolysis in human blood with air admixture is underway to expand the data basis for interpretation, and help to support or refute the present findings.

## Limitations

We used water as the single-phase fluid in this investigation after testing a mixture of glycerol and water with a viscosity similar to that of blood. In a preliminary test, this fluid behaved similarly to pure water. Therefore, for practical reasons, water was the best choice under the given experimental conditions.

Air and fluid aspiration was not studied due to the fact that the MRI technique will result in total signal cancellation under these circumstances.

The use of blood for our study would not have been justifiable for ethical reasons (human blood reserves are scarce), theoretical reasons (animal blood has different flow properties) and cost reasons (10.5 L of blood would have been required to fill the experimental set-up).

In contrast to blood, water is a single-phase Newtonian fluid, and the flow in the cardiotomy suction circuit is not single-phase but multi-phase, best described as a “slug flow”. Particularly in industrial process engineering, nuclear power plant cooling and other heat and mass transfer processes, multiphase flows are widespread and now adequately understood [109, 127, 128]. The chaotic nature of these multiphase flow phenomena is one of the problems in modelling [127]. Similar to the CFD simulation of turbulence with different assumptions and statistical parameters, a “cell unit” model has been described for “slug flow” [128]. Currently, we are not aware of any experimental work in this area for medical applications.

Computational Fluid Dynamics (CFD) simulation appears to be the method of choice to further elucidate these conditions, as no single measurement technique can adequately describe these complex flows. However, we have not yet tackled this task because the computational time, CFD capabilities, and resources, as well as our current theoretical knowledge of fluid dynamics, exceed the requirements.

## Conclusion

We aimed to detect turbulence in cardiotomy suction heads using MRI. We were led by the observation, that turbulence may be a possible source of blood damage. Using MRI, we were able to show that different surgical suction head geometries produce different degrees of turbulence under the same controlled flow conditions. Provided that turbulence leads to increased destruction of blood components, we conclude that optimized suction head geometry may be a valuable factor in maintaining the integrity of red blood cells. Therefore, understanding and optimizing the flow characteristics of different suction head designs is essential to reduce avoidable blood trauma and hence the need for allogenic blood transfusion during surgical procedures. However, due to the wide variety of requirements in specialized surgical procedures, there is no “one size fits all” solution for surgical suction heads.

## Data Availability

The research data associated with this article are included within the article.
